# Characterization of an Activated Metabolic Transcriptional Program in Hepatoblastoma Tumor Cells Using scRNA-seq

**DOI:** 10.3390/ijms252313044

**Published:** 2024-12-04

**Authors:** Claudia Monge, Raquel Francés, Agnès Marchio, Pascal Pineau, Christophe Desterke, Jorge Mata-Garrido

**Affiliations:** 1 Unité Organisation Nucléaire et Oncogenèse, INSERM U993, Institut Pasteur, Université Paris Cité, 75015 Paris, France; claudia.monge@pasteur.fr (C.M.); agnes.marchio@pasteur.fr (A.M.); pascal.pineau@pasteur.fr (P.P.); 2 Energy & Memory, Brain Plasticity Unit, CNRS, ESPCI Paris, PSL Research University, 75006 Paris, France; raquel.frances@espci.fr; 3 Faculté de Médecine du Kremlin Bicêtre, University Paris-Sud, Université Paris-Saclay, 94270 Le Kremlin-Bicêtre, France

**Keywords:** hepatoblastoma, epigenetics, one-carbon, metabolism, cancer, DNA methylation

## Abstract

Hepatoblastoma is the most common primary liver malignancy in children, with metabolic reprogramming playing a critical role in its progression due to the liver’s intrinsic metabolic functions. Enhanced glycolysis, glutaminolysis, and fatty acid synthesis have been implicated in hepatoblastoma cell proliferation and survival. In this study, we screened for altered overexpression of metabolic enzymes in hepatoblastoma tumors at tissue and single-cell levels, establishing and validating a hepatoblastoma tumor expression metabolic score using machine learning. Starting from the Mammalian Metabolic Enzyme Database, bulk RNA sequencing data from GSE104766 and GSE131329 datasets were analyzed using supervised methods to compare tumors versus adjacent liver tissue. Differential expression analysis identified 287 significantly regulated enzymes, 59 of which were overexpressed in tumors. Functional enrichment in the KEGG metabolic database highlighted a network enriched in amino acid metabolism, as well as carbohydrate, steroid, one-carbon, purine, and glycosaminoglycan metabolism pathways. A metabolic score based on these enzymes was validated in an independent cohort (GSE131329) and applied to single-cell transcriptomic data (GSE180665), predicting tumor cell status with an AUC of 0.98 (sensitivity 0.93, specificity 0.94). Elasticnet model tuning on individual marker expression revealed top tumor predictive markers, including FKBP10, ATP1A2, NT5DC2, UGT3A2, PYCR1, CKB, GPX7, DNMT3B, GSTP1, and OXCT1. These findings indicate that an activated metabolic transcriptional program, potentially influencing epigenetic functions, is observed in hepatoblastoma tumors and confirmed at the single-cell level.

## 1. Introduction

Hepatoblastoma is the most common primary liver malignancy in children, accounting for approximately 1% of all pediatric cancers and 80% of liver cancers in children under the age of five [1,2]. Despite being a rare disease, hepatoblastoma has garnered significant attention due to its aggressive nature and the challenges it poses in pediatric oncology. The etiology of hepatoblastoma remains largely unknown, although it is thought to involve a combination of genetic, environmental, and developmental factors of undefined importance [2]. The survival rate for patients with localized hepatoblastoma has improved with advances in surgical techniques and chemotherapy; however, the prognosis for those with metastatic or recurrent disease remains poor [3,4,5,6]. This underscores the urgent need to elucidate the molecular mechanisms underlying hepatoblastoma development and progression to identify novel therapeutic targets.

The liver is a central hub for metabolic processes, including gluconeogenesis, glycolysis, fatty acid oxidation, and metabolite detoxification [7]. These metabolic pathways are tightly regulated to maintain homeostasis and support the liver’s diverse physiological functions. In the context of liver cancer, including hepatoblastoma, metabolic reprogramming is a well-recognized phenomenon [8,9,10,11]. Cancer cells often undergo a metabolic shift known as the Warburg effect, characterized by increased glycolysis and lactate production even in the presence of sufficient oxygen [12,13]. This shift supports rapid cell proliferation by providing the necessary building blocks for biomass production and by maintaining redox balance [14,15].

In hepatoblastoma tumors, Glypican 3 (GPC3) is a frequently overexpressed cell surface heparan sulfate proteoglycan [16]. GPC3 in the HEPG2 hepatoblastoma cell line regulates MAPK and Wnt/β-catenin signaling [17]. Beta-catenin is frequently mutated in this pathological context and is associated with the hepatic stem-like phenotype of hepatoblastoma [18]. Understanding how these metabolic pathways are regulated in hepatoblastoma can provide insights into the disease’s pathogenesis and identify potential metabolic vulnerabilities that can be targeted therapeutically.

Epigenetics refers to heritable changes in gene expression that do not involve alterations in the DNA sequence. These changes are often mediated by modifications such as DNA methylation, histone modifications, and non-coding RNAs [19]. DNA methylation, in particular, plays a critical role in regulating gene expression and maintaining cellular identity. It is mediated by a family of enzymes known as DNA methyltransferases (DNMTs), including DNMT1, DNMT3A, and DNMT3B, which add methyl groups to cytosine residues in DNA, typically leading to gene silencing [20]. Hepatoblastoma, like other cancers, exhibits widespread epigenetic dysregulation, including abnormal DNA methylation. The role of DNMTs in hepatoblastoma is of particular interest, as these enzymes can modulate the expression of genes involved in key oncogenic pathways and metabolic processes. Investigating the epigenetic landscape of hepatoblastoma can reveal critical insights into how DNA methylation and other epigenetic modifications contribute to tumor development and progression [21].

Oxidative stress, characterized by an imbalance between the production of reactive oxygen species (ROS) and the cell’s antioxidant defenses, plays a pivotal role in cancer biology. Mitochondria, the cell’s powerhouse, are both a major source and target of ROS [22]. In liver cancer, including hepatoblastoma, mitochondrial dysfunction and oxidative stress are common features that drive tumorigenesis. ROS can induce DNA damage, promote genetic instability, and activate signaling pathways that support cancer cell survival and proliferation [23,24].

Understanding the interplay between oxidative stress, mitochondrial function, and the metabolic and epigenetic landscape of hepatoblastoma can provide a comprehensive view of the molecular mechanisms driving this disease [24,25].

Given the importance of metabolic and epigenetic regulation in liver physiology and cancer, our study aims to explore these aspects in hepatoblastoma using single-cell RNA sequencing (scRNAseq). By profiling the transcriptomes of individual cells within hepatoblastoma tumors, we can uncover the cellular heterogeneity and identify distinct metabolic and epigenetic states. Specifically, we aim to elucidate the relationship between the metabolic status of hepatoblastoma cells and their tumorigenic status, as well as the role of DNMT-mediated DNA methylation in regulating these processes.

## 2. Results

### 2.1. Differential Expressed Enzymes in Tumors as Compared to Normal Adjacent Liver Tissues from Hepatoblastoma Human Samples

**Figure 1:**
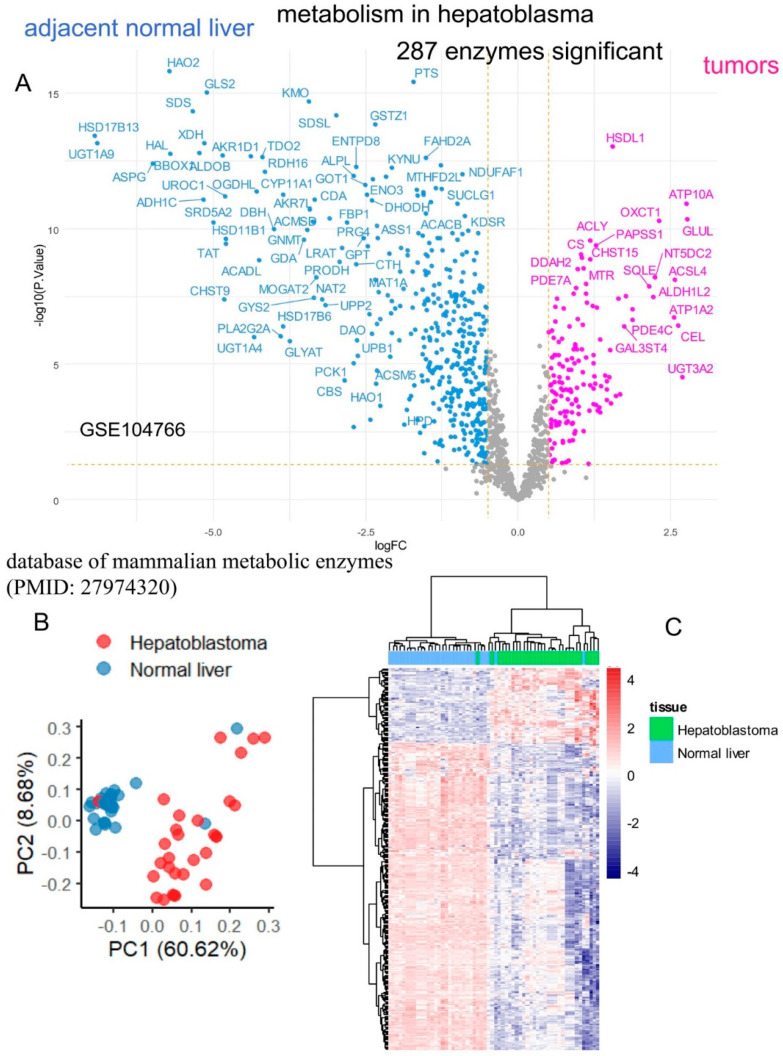
Differentially expressed enzymes in tumors compared to normal adjacent liver tissues from human hepatoblastoma samples: (**A**) Volcano plot of differentially expressed enzymes in the GSE104766 transcriptome dataset; (**B**) Principal component analysis of 287 differentially expressed enzymes; (**C**) Unsupervised clustering (Euclidean distances) with an expression heatmap of the 287 differentially expressed enzymes.

The RNA-sequencing dataset GSE104766 [26], which includes samples from human hepatoblastoma tumors and adjacent liver tissue, was preprocessed using edgeR normalization, voom transformation, low-expression filtering, and quantile normalization (Figure S1). The normalized data were subsequently analyzed using supervised methods focused on the expression of mammalian metabolic enzymes. Differential gene expression analysis between tumor samples and adjacent liver tissue identified 287 significantly regulated enzymes (Table S1, Figure 1A). Principal component analysis (PCA) stratified the majority of samples effectively based on their tissue origin (Figure 1B). Similarly, unsupervised clustering (Euclidean distances, Ward.D2 method) with a heatmap constructed using the expression of the 287 significant enzymes successfully grouped most samples according to their tissue origin (Figure 1C). These findings suggest that hepatoblastoma tumors exhibit a dysregulated metabolic program compared to adjacent liver tissue.

### 2.2. Activated Metabolic Transcriptional Program in Hepatoblastoma Tumors

**Figure 2:**
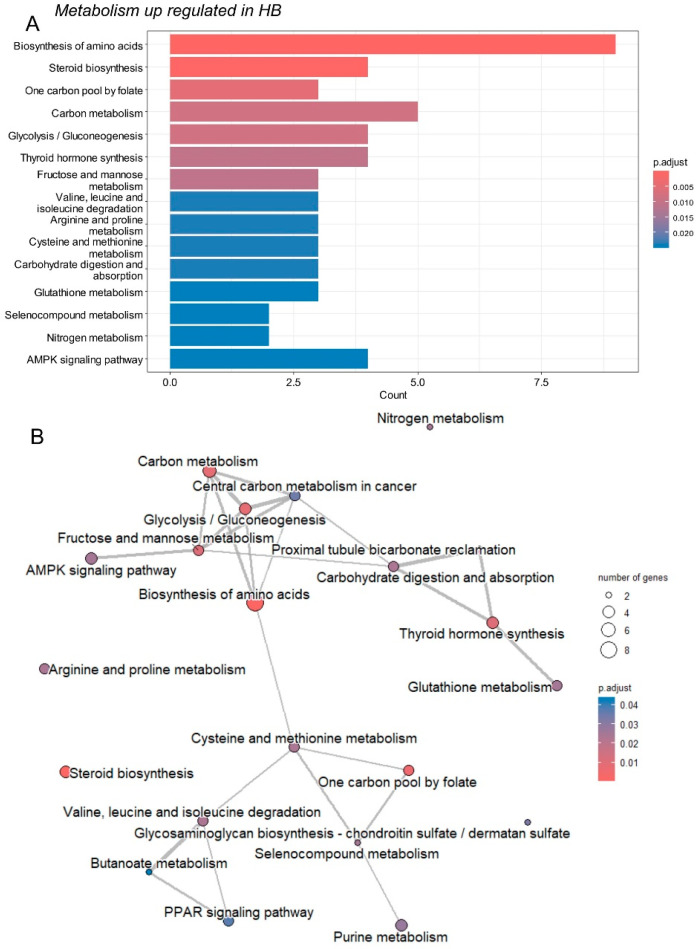
Activated metabolic transcriptional program in hepatoblastoma tumors: GSE104766: (**A**) Bar plot of functional enrichment (KEGG database) performed on 59 enzymes found overexpressed in hepatoblastoma tumors; (**B**) Emaplot of functional enrichment (KEGG database) performed on 59 enzymes found overexpressed in hepatoblastoma tumors.

Hepatoblastoma tumor cells may exhibit stemness properties, as seen in the C2 class, rendering them less differentiated than normal hepatocytes [18]. Consequently, the normal metabolic transcriptional program of hepatocytes is often repressed, as observed in the GSE104766 dataset (Figure 1A) [26]. The analysis focused on metabolic enzymes that were overexpressed in tumors. Among the 287 differentially expressed enzymes identified in the bulk transcriptome analysis, 59 were found to be overexpressed in tumor samples compared to adjacent liver tissue (Figure 1A, Table S1). Functional enrichment analysis of these overexpressed enzymes was performed using the Kyoto Encyclopedia of Genes and Genomes (KEGG) database. Of the 59 overexpressed enzymes, 45 were recognized by KEGG. This enrichment analysis revealed significant involvement of pathways associated with amino acid, carbohydrate, steroid, one-carbon, purine, and glycosaminoglycan metabolisms (Figure 2, Table 1).

### 2.3. Activated Metabolic Network in Hepatoblastoma Tumors

**Figure 3:**
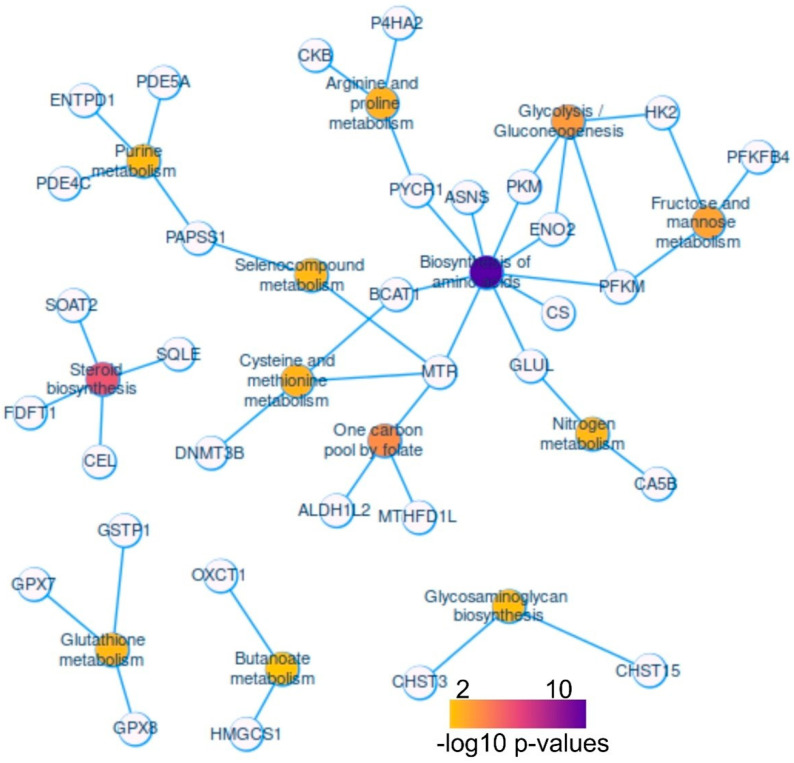
Activated metabolic network in human hepatoblastoma tumors: GSE104766, Functional enrichment network of metabolic enzyme actors found over expressed in human hepatoblastoma tumors (enrichment on KEGG database), color scale from yellow to purple represent the negative logarithm base 10 of the enriched *p*-values.

Using KEGG enrichment analysis on the metabolic enzymes overexpressed in hepatoblastoma tumors, a functional enrichment network was constructed using the Cytoscape application. This network revealed high connectivity, with a central focus on amino acid biosynthesis metabolism (Figure 3). The amino acid biosynthesis pathway featured nine direct connections with enzymes: pyrroline-5-carboxylate reductase 1 (PYCR1), asparagine synthetase (glutamine-hydrolyzing) (ASNS), pyruvate kinase M1/2 (PKM), enolase 2 (ENO2), phosphofructokinase, muscle (PFKM), citrate synthase (CS), glutamate-ammonia ligase (GLUL), 5-methyltetrahydrofolate-homocysteine methyltransferase (MTR), and branched-chain amino acid transaminase 1 (BCAT1). Amino acid biosynthesis was the most significantly enriched pathway (FDR q-value = 7.40 × 10^−9^, Table 1). Notably, DNA methyltransferase 3 beta (DNMT3B) plays a role in cysteine and methionine metabolism within this network (Figure 3).

The second most significantly enriched pathway, steroid metabolism (FDR q-value = 1.03 × 10^−4^, Table 1), was also identified in the KEGG network, involving four enzymes: squalene epoxidase (SQLE), carboxyl ester lipase (CEL), farnesyl-diphosphate farnesyltransferase 1 (FDFT1), and sterol O-acyltransferase 2 (SOAT2) (Figure 3).

The one-carbon pool of folate pathway was represented by three enzymes (FDR q-value = 3.39 × 10^−3^, Table 1): 5-methyltetrahydrofolate-homocysteine methyltransferase (MTR), aldehyde dehydrogenase 1 family member L2 (ALDH1L2), and methylenetetrahydrofolate dehydrogenase (NADP^+^-dependent) 1-like (MTHFD1L).

Additionally, carbohydrate metabolism was represented by several key enzymes, such as hexokinase 2 (HK2) and 6-phosphofructo-2-kinase (PFKFB4), some of which overlap with amino acid biosynthesis metabolism (e.g., PKM, PFKM, and ENO2) (Figure 3).

**Figure 4:**
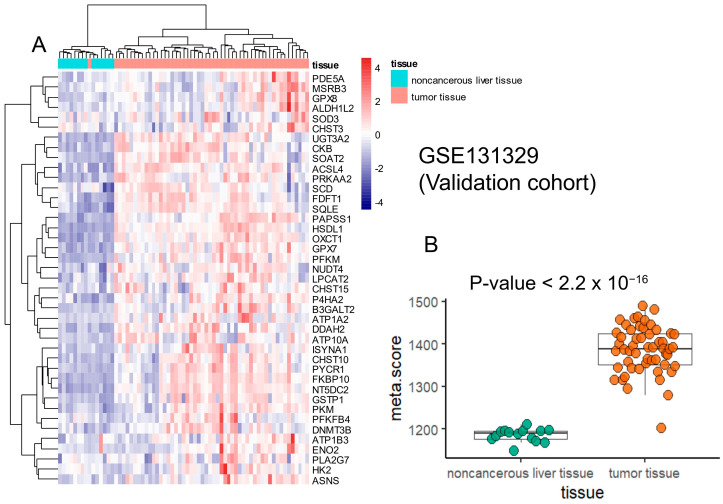
Validation of the metabolic signature on an external cohort of hepatoblastoma transcriptomes: dataset GSE131329. (**A**) Unsupervised clustering and expression heatmap of the metabolic score signature. (**B**) Calculation of the metabolic score according to tissue groups: the *p*-value evaluating the difference between the two tissue groups was obtained by a two-sided Student’s *t*-test.

In the validation cohort of hepatoblastoma tumor transcriptomes (GSE131329), the metabolic signature effectively stratified tumor samples from non-cancerous liver tissue through unsupervised clustering (Euclidean distances, Figure 4A). Additionally, in this validation cohort, the metabolic score was calculated and found to be significantly higher in hepatoblastoma compared to non-cancerous liver tissue (two-sided *t*-test, *p*-value < 2.2 × 10^−16^, Figure 4B). These findings suggest that our hepatoblastoma metabolic signature and metabolic score are robust, as they were successfully validated in an independent external cohort of hepatoblastoma transcriptomes.

### 2.4. High Level of Metabolic Score at Single Cell Level in Tumor Cells from Hepatoblastoma

**Figure 5:**
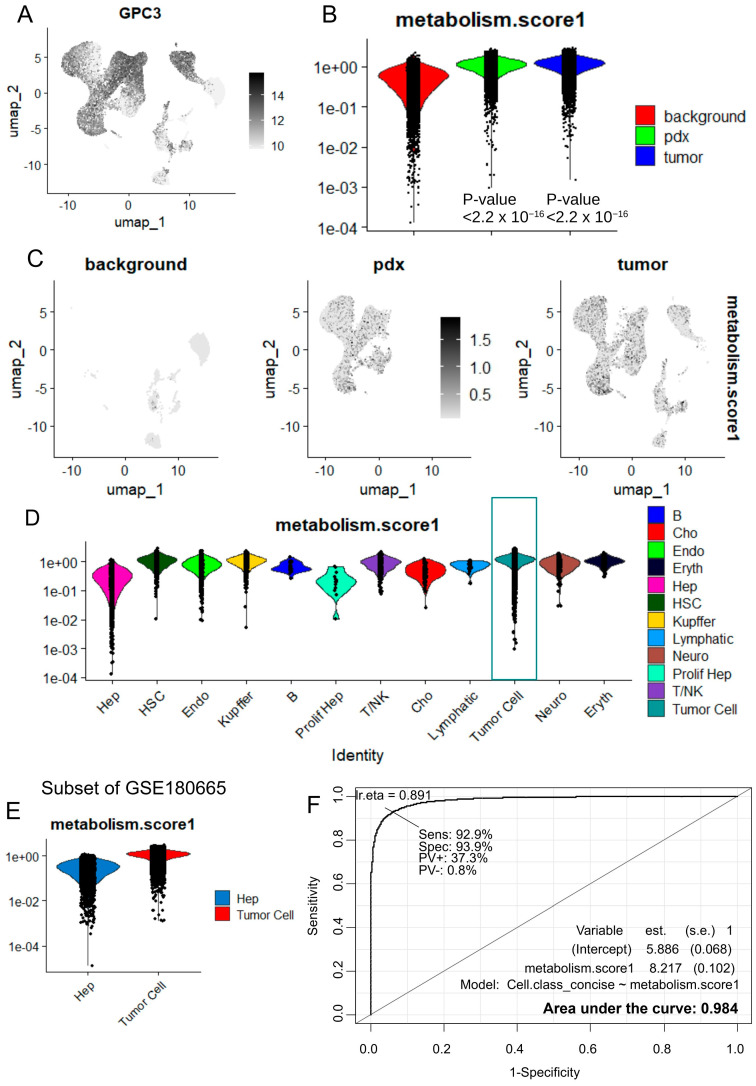
High level of metabolic score at the single-cell level in tumor cells from hepatoblastoma: GSE180665 (**A**) UMAP dimensional reduction with expression of Glypican 3 (GPC3). (**B**) Violin plot of the metabolic score across sample groups (*p*-value, two-sided *t*-test). (**C**) Feature plot of metabolic score, split by sample types (computed on 59 HB-activated enzymes) on UMAP dimensional reduction. (**D**) Violin plot of metabolic score stratified by cell types: normal hepatocytes (Hep) in pink. (**E**) Violin plot of metabolic score comparing normal hepatocytes in blue (Hep) versus tumor cells (PDX + human tumors) by scRNA-seq. (**F**) ROC curve and area under the curve for metabolic score in predicting hepatoblastoma tumor cells as compared to normal hepatocytes.

To validate the metabolic signature observed in bulk RNA sequencing, the single-cell expression of these tumor upregulated enzymes was investigated in the single-cell RNA sequencing dataset GSE180665 [27]. This dataset comprises seven experiments from 10x Genomics single-cell RNA-seq performed on three hepatoblastoma tumors, two patient-derived xenotransplant models (PDX), and two liver-adjacent tissues. Sequencing counts from the seven experiments were integrated into a single-cell Seurat object to perform dimensionality reduction by principal component analysis (Figure S2A), followed by UMAP (Figure S2B). Cells were annotated according to their original cell annotations (Figure S2B). Sample identity projection on the UMAP dimensional reduction revealed a heterogeneous integration of the seven samples (Figure S2C). Harmony integration was performed on this single-cell object, split by sample origin. Harmony integration effectively stratified the original cell annotations (Figure S2D) and allowed for a good integration of the samples according to their origins (Figure S2E). These results suggest that Harmony integration correctly integrated the seven experiments of the GSE180665 dataset, with satisfactory stratification of the original cell-type annotations. A second UMAP dimensional reduction was performed on the Harmony-post-integrated single-cell object. This dimensional reduction correctly distributed the samples (Figure S3A) according to their sample groups (tumor, PDX, liver; Figure S3B), original cell clustering (Figure S3C), and original cell-type annotations (Figure S3D). Glypican 3, a known marker expressed in hepatoblastoma tumors [28], was predominantly expressed in hepatoblastoma tumor cells but not in normal hepatocytes (Figure 5A). Using the “AddModuleScore” function in Seurat [29], a metabolic score was computed based on the expression of the 59 enzymes found to be overexpressed in hepatoblastoma tumors (Table S1). As expected, this metabolic score was found to be significantly higher in hepatoblastoma tumor cells compared to other cell types present in the samples (Figure 5B–D and Table 2).

### 2.5. Increase of Metabolic Score Is Highly Predictive of Tumor Cell Status in Single Cell Transcriptome from Hepatoblastoma

The GSE180665 dataset was refined to directly compare normal hepatocytes with tumor cells in a new subset. A higher metabolic score was observed in tumor cells (both human and PDX) compared to normal hepatocytes (Figure 5E) (two-sided *t*-test, *p*-value < 2.2 × 10^−6^, Figure 5B). The ROC curve computed for the metabolic score to predict tumor cell identity versus normal hepatocytes showed an area under the curve (AUC) of 0.984 (Figure 5F). The metabolic score predicted tumor cell status with a sensitivity of 0.93 and a specificity of 0.94 (Figure 5F). These findings suggest that the metabolic score is highly predictive of tumor cell status at the single-cell level.

### 2.6. Metabolism Score Harbored Greater Value in G1 Quiescent Tumor Cells Derived from Hepatoblastoma Tumoroids

**Figure 6:**
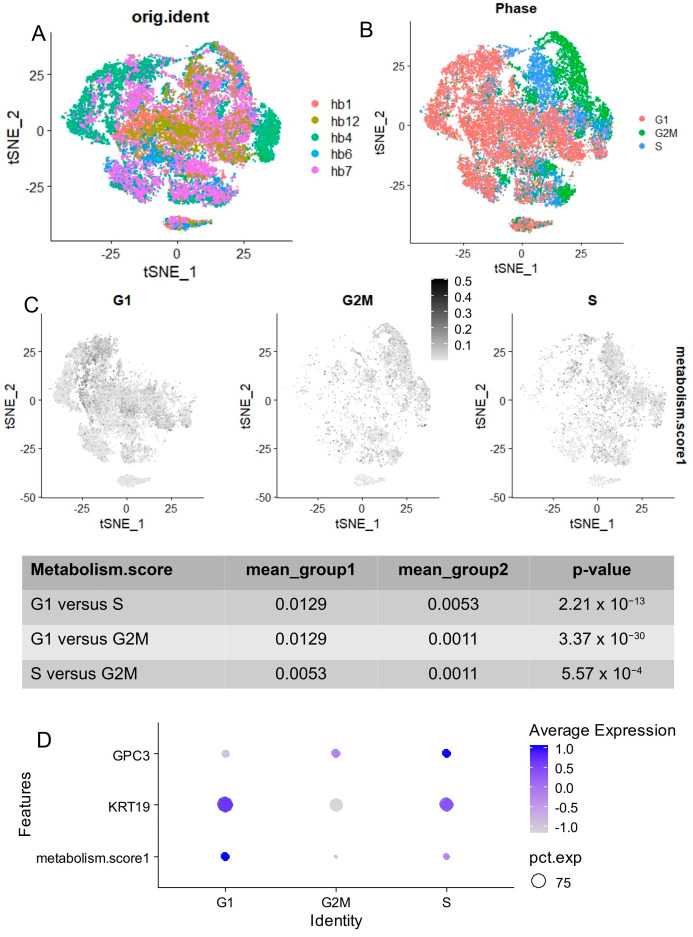
Higher levels of metabolic score quantified in G1 quiescent cells derived from human hepatoblastoma tumoroids: scRNA-seq dataset GSE233923. (**A**) t-SNE dimensional reduction of scRNA-seq transcriptomes from five human hepatoblastoma tumoroids post canonical correlation integration. (**B**) t-SNE dimensional reduction with cell cycle phase prediction (G1: Gap1, S: DNA synthesis, G2M: Gap2/Mitosis). (**C**) Metabolic score quantification on t-SNE dimensional reduction with stratification by cell cycle phases (*p*-values in tables were obtained by a two-sided Student’s *t*-test in the table). (**D**) Dot plot stratified by cell cycle phases for quantification of metabolic score, KRT19, and GPC3 expression.

The quantification of single-cell heterogeneity based on the metabolic score was assessed using a human hepatoblastoma validation dataset (GSE233923). Five single-cell transcriptome experiments performed on five distinct tumoroids were integrated using canonical correlation (Figure 6A). Cell cycle phase prediction was conducted on this dataset with Seurat R-package performing regression on cell cycle markers provided in the package (Figure 6B). The metabolic score, when stratified by predicted cell cycle phases, was found to be significantly higher in HB tumoroid cells predicted to be in the G1 quiescent phase (Gap1) compared to cells in the G2M and S phases (Figure 6C). These G1-phase tumoroid cells, which had a higher metabolic score, also showed high expression of the stem cell marker KRT19 and low expression of Glypican 3 (Figure 6D).

### 2.7. Importance of over Expressed Metabolic Markers to Predict Tumor Cell Status in scRNAseq of Hepatoblastoma Samples

**Figure 7:**
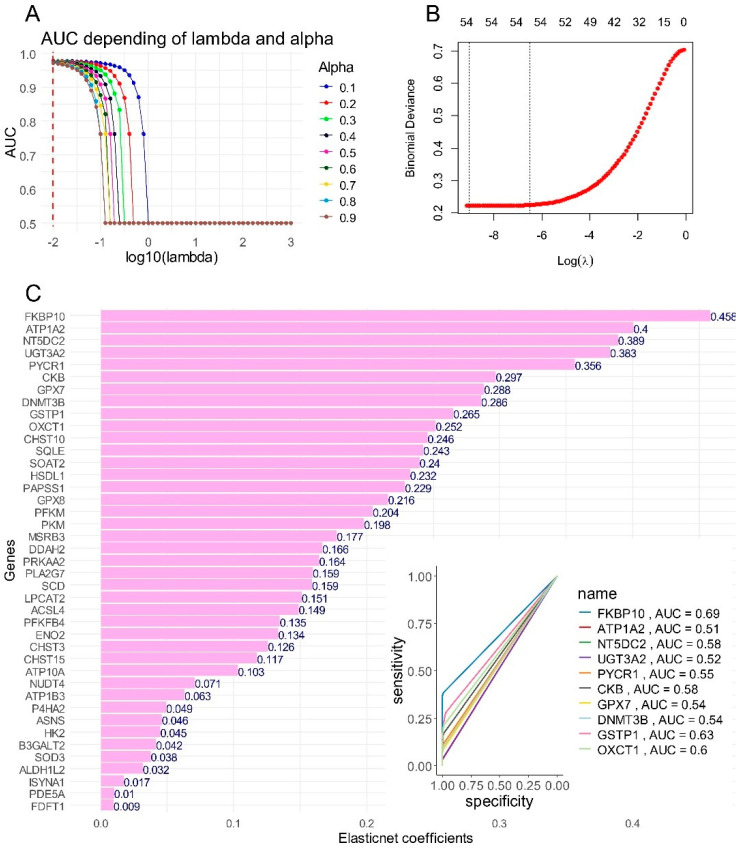
Importance of overexpressed metabolic markers to predict tumor cell status in scRNA-seq of hepatoblastoma samples: GSE180665. (**A**) Alpha and lambda parameter tuning from the ElasticNet model with a 70/30 training/validation split of the GSE180665 dataset: tumor cells versus normal hepatocyte status prediction. (**B**) Best lambda estimation for the alpha parameter fixed at 0.1 in the ElasticNet model. (**C**) Positive ElasticNet coefficients for 41 metabolic markers expressed in HB tumor cells at the single-cell level, with individual area under the curve (AUC) values for the ten best predictive enzymes.

The metabolic score has been shown to be highly predictive of tumor cell status at the single-cell level. Using the scRNA-seq dataset GSE180665, an artificial intelligence model based on ElasticNet was developed to determine the individual contribution of each enzyme involved in calculating the metabolic score. The single-cell data, restricted to tumor cells and hepatocytes, was split into training and validation sets in a 70/30 ratio. Tuning of the alpha and lambda parameters in the ElasticNet model to predict tumor cell status was performed (Figure 7A), yielding the best area under the curve (AUC = 0.975) for an alpha value of 0.1 (Figure 7B). In the second step, the optimal ElasticNet model was fitted using this alpha value (Figure S4A). ElasticNet coefficient estimation was carried out by determining the coefficient of variation within the optimal lambda range (Figure 7B). Of the 59 enzymes overexpressed in hepatoblastoma tumors, 41 were assigned positive coefficients in the ElasticNet model, indicating their role in predicting tumor cell status at the single-cell level (Figure 7C). The combined expression of these 41 enzymes continued to predict tumor cell status with an AUC of 0.965, a sensitivity of 0.88, and a specificity of 0.95 (Figure S4B). This optimized ElasticNet model (alpha = 0.1, AUC = 0.975) allowed for the ranking of markers based on their importance in predicting tumor cell status. The top ten markers were: FKBP prolyl isomerase 10 (FKBP10), ATPase Na^+^/K^+^ transporting subunit alpha 2 (ATP1A2), 5′-nucleotidase domain containing 2 (NT5DC2), UDP glycosyltransferase family 3 member A2 (UGT3A2), pyrroline-5-carboxylate reductase 1 (PYCR1), creatine kinase B (CKB), glutathione peroxidase 7 (GPX7), DNA methyltransferase 3 beta (DNMT3B), glutathione S-transferase pi 1 (GSTP1), and 3-oxoacid CoA-transferase 1 (OXCT1).

## 3. Discussion

In hepatoblastoma, metabolic reprogramming is particularly relevant due to the liver’s intrinsic metabolic functions. Enhanced glycolysis, glutaminolysis, and fatty acid synthesis have been linked to the proliferation and survival of hepatoblastoma cells [11,16,17,18]. In the present work, the upregulation of the metabolic enzyme program was investigated in hepatoblastoma tumors at both the tissue and single-cell levels. We confirmed major alterations in carbohydrate metabolism, which share some enzymes with amino acid biosynthesis metabolism, such as PKM, PFKM, and ENO2. PKM2 is an alternative splice isoform of the PKM gene. The M2 pyruvate kinase (PKM2) isoform is upregulated in most cancers and plays a crucial role in regulating the Warburg effect, which is characterized by the preference for aerobic glycolysis over oxidative phosphorylation for energy metabolism. Antisense oligonucleotide-based PKM splice switching has been proposed as a targeted therapy for liver cancer [30]. During hepatocellular carcinoma, ZEB1 has been shown to enhance the Warburg effect, facilitating tumorigenesis and metastasis by transcriptionally activating PFKM [12]. In amino acid biosynthesis, GLUL and ASNS have been shown to correlate with overall survival during hepatoblastoma. Hepatoblastoma samples exhibited strong GLUL expression and glutamine synthesis, generally as a result of CTNNB1 mutations. Glutamine depletion inhibited proliferation and cell viability in embryonal hepatoblastoma cell lines [28]. Furthermore, CTNNB1 mutations play a broader role in liver cancer by activating the Wnt/β-catenin pathway, which is further influenced by the overexpression of Glypican 3 (GPC3). GPC3 modulates MAPK and Wnt/β-catenin signaling in hepatoblastoma, as observed in the HEPG2 cell line, highlighting it as a potential therapeutic target. Additionally, the Wnt pathway’s link to cancer stem cells (CSCs), marked by Lgr5, emphasizes Lgr5’s role as a biomarker in cancer progression and drug resistance. These CSCs drive tumor initiation, growth, and resistance to therapy, with Lgr5 supporting proliferation and self-renewal through Wnt/β-catenin signaling. Targeting Lgr5+ CSCs offers therapeutic potential, but single-target therapies alone may not suffice to prevent recurrence. Drug screening targeting Lgr5-related pathways could aid in personalized therapy development, while Lgr5 may serve as a candidate for antibody-based or drug delivery therapies [31,32]. This integrated perspective may ultimately enhance therapeutic approaches not only for hepatoblastoma but also for other cancers involving similar pathways.

In the metabolic enrichment network, steroid metabolism was also highlighted as an isolated subnetwork implicating SQLE, SOAT2, FDFT1, and CEL. Carboxyl ester lipase is implicated in reverse cholesterol transport [33] and confers susceptibility to alcoholic liver cirrhosis [34]. Squalene epoxidase (SQLE) is known to promote the growth and migration of hepatocellular carcinoma cells [35]. During zebrafish embryogenesis, Sterol O-Acyltransferase 2 contributes to yolk cholesterol trafficking [36].

During our work, we observed a strong interconnection between metabolism and epigenetics, specifically in the regulation of DNA methylation. DNMT3B, a DNA methyltransferase, is a key player in this epigenetic machinery and catalyzes the addition of methyl groups to DNA, influencing gene expression patterns [37]. This enzyme not only affects gene silencing but also contributes to metabolic reprogramming by modulating the expression of metabolic genes [37]. Thus, DNMT3B is likely involved in coordinating metabolic and epigenetic adaptations that enable cancer cells to sustain proliferation under diverse conditions. During hepatoblastoma, a general disruption in the expression of genes from the epigenetic machinery was observed, mainly upregulation of UHRF1, TET1, and TET2, in association with an enrichment of 5 hmC content. These alterations support a model of active demethylation by TETs in hepatoblastoma, probably during early stages of liver development, which, in combination with UHRF1 overexpression, would lead to DNA hypomethylation and an increase in overall 5 hmC content [38]. UHRF1 (Ubiquitin-like with PHD and RING Finger Domains 1) plays a vital role in maintaining DNA methylation patterns in cancer cells, supporting both epigenetic regulation and metabolic reprogramming [39]. UHRF1 coordinates with DNMT1 to ensure DNA methylation during cell division, a key process for silencing tumor suppressor genes and promoting oncogenesis. Beyond its canonical role in DNA methylation, UHRF1 affects metabolic pathways by altering gene expression linked to glycolysis and the tricarboxylic acid cycle, both crucial for meeting the high metabolic demands of proliferating cancer cells [39]. This dual role underlines UHRF1′s potential as a therapeutic target in metabolic and epigenetic dysregulation in cancer.

During the development of metabolic dysfunction associated with liver cancer, associations were observed between rare and common germline variants in one-carbon metabolism and DNA methylation genes [40]. The KEGG enrichment network of hepatoblastoma tumor cells showed enrichment in one-carbon metabolism enzymes, regulating the pool of folates. Depletion of folates is connected to the aggressiveness of the cancer phenotype [41], and the folate pool can be dependent on purine nucleotide biosynthesis. Hepatoblastoma tumor cells exhibited upregulation of purine metabolism through enhanced expression of ENTPD1, PDE4C, PDE5A, and PAPSS1. Disruption of ENTPD1 (Cd39) perturbs metabolism (purinergic signaling) during liver development [42]. The use of phosphodiesterase inhibitors during chronic liver injury and metabolic diseases may impact cyclic AMP (cAMP) signaling, particularly in the regulation of fatty acid (FA) β-oxidation and pro-inflammatory polarization of tissue-resident lymphocytes [43]. In the HEpG2 cell line, PAPSS1/2 knockdown significantly activated farnesoid X receptor (FXR), retinoid-related orphan receptor, and pregnane X receptor-responsive reporters, with treatment using the FXR agonist GW4064 [44].

One-carbon metabolism deregulation in hepatoblastoma tumors implicated 5-methyltetrahydrofolate-homocysteine methyltransferase (MTR), aldehyde dehydrogenase 1 family member L2 (ALDH1L2), and methylenetetrahydrofolate dehydrogenase (NADP^+^-dependent) 1-like (MTHFD1L). Mitochondrial folate-dependent one-carbon (1-C) metabolism converts 1-C donors such as serine and glycine to formate, which is exported and incorporated into the cytoplasmic tetrahydrofolate (THF) 1-C pool [45]. The folate cycle, through the transfer of a carbon unit between tetrahydrofolate and its derivatives in the cytoplasmic and mitochondrial compartments, produces other metabolites that are essential for cell growth, including nucleotides, methionine, and the antioxidant NADPH. The folate cycle enzyme MTHFD1L is known to confer metabolic advantages in hepatocellular carcinoma [46]. During embryonic development, the methylation of DNA and histones drives cell division and regulation of gene expression through epigenesis and imprinting. Folate cycle and methyltransferase enzymes are important actors in methyl transfer processes [47].

At the single-cell level, the machine learning model ranked the top ten mRNAs to predict tumor cell status in hepatoblastoma: FKBP10, ATP1A2, NT5DC2, UGT3A2, PYCR1, CKB, GPX7, DNMT3B, GSTP1, and OXCT1. FKBP10 is a member of the FK506-binding protein (FKBP) family and has been implicated in cancer development [48]. In lung cancer, gain- and loss-of-function assays show that FKBP10 boosts cancer growth and stemness via its peptidyl-prolyl-cis-trans-isomerase (PPIase) activity. FKBP10 also interacts with ribosomes, and its downregulation leads to a reduction in translation elongation at the beginning of open reading frames (ORFs), particularly upon the insertion of proline residues [49]. ATP1A2 has been shown to be highly expressed in platinum-resistant ovarian cancer [50]. In hepatocellular carcinoma, NT5DC2 promotes tumor cell proliferation by stabilizing EGFR [51]. UGT3A2, a UDP-glycosyltransferase (UGT), is implicated in the detoxification of exogenous polycyclic aromatic hydrocarbons (PAHs) [52]. Pyrroline-5-carboxylate reductase (PYCR1) participates in mitochondrial proline metabolism reprogramming and promotes liver tumorigenesis [53]. Creatine kinase B (CKB) suppresses ferroptosis by phosphorylating GPX4 [54]. GPX7, along with GPX4, is known to be overexpressed in hepatocellular carcinoma tissues [55]. The GSTP1 gene is preceded by a large CpG-rich region that is frequently affected by methylation during cancer [56]. 3-oxoacid CoA-transferase 1 (OXCT1), a rate-limiting ketolytic enzyme whose expression is suppressed in normal adult liver tissues, is re-induced by serum starvation-triggered mTORC2-AKT-SP1 signaling in HCC cells [57]. The top ten enzymes in the hepatoblastoma metabolic score confirmed the potential reprogramming of these tumor cells.

This work highlights the complex interplay between metabolic reprogramming and epigenetic dysregulation in hepatoblastoma, particularly through the overexpression of DNMT3B and the involvement of one-carbon metabolism in tumor cells. It points to potential therapeutic targets and biomarkers for this aggressive pediatric cancer, providing novel insights into the molecular underpinnings of hepatoblastoma and underscoring the value of integrated single-cell analyses in understanding cancer biology. The claim of a “complex interplay” between metabolism and epigenetic regulation in the context of cancer biology is well-supported by a growing body of literature, even though this specific study lacks direct experimental evidence to substantiate the relationship. Metabolism and epigenetic processes are closely interconnected, with metabolites such as acetyl-CoA, S-adenosylmethionine (SAM), and α-ketoglutarate directly influencing key epigenetic modifications. For example, acetyl-CoA serves as a substrate for histone acetylation [58], while SAM is the primary methyl donor for DNA and histone methylation [59,60]. Similarly, enzymes such as DNMT3B or UHRF1 are dependent on metabolic byproducts for their activity [61,62,63]. Changes in metabolic pathways, commonly observed in tumor cells, can alter the availability of these metabolites, thereby influencing epigenetic marks and gene expression. Therefore, the upregulation of both metabolic genes and epigenetic regulators in hepatoblastoma suggests a broader regulatory network that coordinates metabolic reprogramming with epigenetic changes to support tumor growth. While this study did not experimentally verify the direct connections, existing research supports the likelihood of such crosstalk, as metabolic shifts can drive epigenetic alterations, promoting oncogenesis and tumor progression.

For perspectives, in a less invasive context, it would be interesting to invest in the detection of these enzymes and their metabolites in the blood of patients in order to refine their diagnosis. But also it would be interesting to link this metabolic signature to the prognosis of patients with hepatoblastoma.

Future research on the functional analysis of these pathways in the liver is needed to provide more conclusive evidence, including knock-out or overexpression mouse models of these epigenetic/metabolic pathways. In addition, many members of these pathways are druggable, such as DNA methyltransferase inhibitors [64]. This study therefore lays the groundwork for further translational studies or clinical trials.

## 4. Materials and Methods

### 4.1. Public Dataset of RNA-Sequencing Performed on Human Hepatoblastoma Tissues

#### 4.1.1. Training Cohort

RNA-sequencing performed on tumor and adjacent liver tissue from human hepatoblastoma was investigated through Gene Expression Omnibus (GEO) [65,66] dataset GSE104766 [26]. Original RNA-seq counts from this study was downloaded at the following web address: https://www.ncbi.nlm.nih.gov/geo/query/acc.cgi?acc=GSE104766 (accessed on 1 August 2024). This cohort comprised 30 tumor samples and 30 noncancerous liver tissue samples.

#### 4.1.2. Validation Cohort

GSE131329 transcriptome dataset [67] was downloaded on Gene Expression Omnibus with GEOquery R-package version 2.70.0 in R software environment version 4.3.3. This microarray dataset is available at the following address: https://www.ncbi.nlm.nih.gov/geo/query/acc.cgi?acc=GSE131329 (accessed on 18 September 2024). It comprised experiments performed with [HuGene-1_0-st] Affymetrix Human Gene 1.0 ST Array microarray technology corresponding to annotation platform GPL6244 available at the following address: https://www.ncbi.nlm.nih.gov/geo/query/acc.cgi?acc=GPL6244 (accessed on 18 September 2024). This transcriptome dataset comprised experiments performed on 53 hepatoblastoma tissues and 14 noncancerous liver tissue samples.

### 4.2. Public Dataset of Single Cell RNA_Sequencing Performed on Hepatoblastoma Samples

Single cell RNA-sequencing experiments from GEO dataset GSE180665 [27] were downloaded at the following web address: https://www.ncbi.nlm.nih.gov/geo/query/acc.cgi?acc=GSE180665 accessed on 1 August 2024). This dataset comprised seven scRNAseq 10xGenomics experiments performed on: three hepatoblastoma tumors, two patient derived xenotransplantation (PDX) models, and two liver adjacent tissue. Raw counts in H5AD format were converted and in single cell experiment object with zellkonverter R-bioconductor package [68]. Original cell annotation was added to the metadata of the scRNAseq object after its transformation in Seurat object [29].

### 4.3. Mammaliam Metabolic Transcriptional Program

Mammalian Metabolic Enzyme Database [69], was downloaded at the following web address: https://esbl.nhlbi.nih.gov/Databases/KSBP2/Targets/Lists/MetabolicEnzymes/MetabolicEnzymeDatabase.html (accessed on 1 August 2024) and annotated with Ensembl Biomart database version 110 [70] through geneconverter R-package accessible at the address: https://github.com/cdesterke/geneconverter (accessed on 1 August 2024).

### 4.4. RNA-Sequencing Analyses

Bioinformatics analyses were performed in R software environment version 4.4.1. RNA-sequencing counts were normalized with edgeR R-bioconductor package version 4.2.0 [71], and normalized count were voom transformed with limma R-bioconductor package version 3.60.3 [72,73]. For batch adjustment, a quantile normalization was applied on voom data with preprocessCore R-bioconductor package version 1.66.0 [74]. Differential expression analysis and transcriptome data visualization: heatmap, principal component analysis, and volcanoplot were performed with transpipe R-package version 1.4 available at the following web address: https://github.com/cdesterke/transpipe14 (accessed on 1 August 2024) [75]. With over expressed enzymes found in hepatoblastoma tumors, a functional enrichment was performed on Kyoto Encyclopedia of Genes and Genomes (KEGG) database [76], clusterProfiler R-bioconductor package version 4.12.2 [77]. A metabolic functional enrichment network was built with Cytoscape standalone application version 3.10.1 [78].

### 4.5. Single Cell RNA-Sequencing Metabolic Score Quantification

Based on single cell expression of the enzyme found over expressed in hepatoblastoma tumors by bulk RNA-sequencing, a metabolic score was computed at single cell level in scRNAseq dataset with “AddModuleScore” Seurat function [29]. ROC curve and area under curve to predict tumor cell status with metabolic score were determined with pROC R-package version 1.18.5 [79].

### 4.6. Validation Dataset of scRNAseq for Human Hepatoblastoma Tumoroids

Single cell transcriptome from five distinct human hepatoblastoma 3D culture tumoroids (dataset GSE233923) was investigated to validate metabolic score according cell cycle phase prediction.

### 4.7. Machine Learning Elasticnet Model on Metabolic Markers

Single cell expression of overexpressed enzymes was extracted from tumor cell and normal hepatocyte in dataset GSE180665 and combined to the corresponding metadata. After data splitting data in training and validation sets (0.7/0.3 ratio), Elasticnet model (tumor cell status binary outcome) was tuning on alpha and lambda parameters with caret R-package version 6.0–94 [80]. Final Elasticnet was fit with best alpha parameter (alpha = 0.1) with glmnet R-package version 4.1–8 [81].

### 4.8. Transcriptomic Metabolic Score

Based on expression of the 41 metabolic enzymes with positive prediction obtained by machine learning a metabolic expression score was computed in microarray cohort GSE131329. The score was obtained by summing the products concerning the expression and ElasticNet coefficients of these enzymatic markers. ElasticNet coefficients were obtained on independent cohort GSE180665.

## 5. Conclusions

This study reveals a complex interplay between metabolic reprogramming and epigenetic dysregulation in hepatoblastoma, as demonstrated through transcriptomic analysis highlighting the overexpression of DNMT3B and the implications of one-carbon metabolism in tumor cells. While these findings provide valuable insights into the molecular underpinnings of hepatoblastoma, it is important to note that our analysis is based solely on transcriptomic data, which may not fully capture the dynamic nature of metabolic pathways or the functional implications of these alterations. Limitations inherent in transcriptomic studies, such as the lack of information on protein expression and post-translational modifications, necessitate caution in translating these findings into therapeutic targets and biomarkers. Nonetheless, this research underscores the potential of integrated single-cell analyses to enhance our understanding of cancer biology and guide future investigations into effective interventions for this aggressive pediatric cancer.

## Figures and Tables

**Table 1:** KEGG functional enrichment of activated metabolic enzymes in tumor cells from hepatoblastoma.

ID	Description	Subcategory	Count	q Value
hsa01230	Biosynthesis of amino acids	Amino acid	9	7.40 × 10^−9^
hsa00100	Steroid biosynthesis	Lipid	4	1.03 × 10^−4^
hsa00670	One carbon pool by folate	Cofactors and vitamins	3	3.39 × 10^−3^
hsa01200	Carbon metabolism	Carbon	5	5.46 × 10^−3^
hsa00010	Glycolysis/Gluconeogenesis	Carbohydrate	4	5.46 × 10^−3^
hsa00051	Fructose and mannose metabolism	Carbohydrate	3	7.25 × 10^−3^
hsa00280	Valine, leucine and isoleucine degradation	Amino acid	3	1.60 × 10^−2^
hsa00330	Arginine and proline metabolism	Amino acid	3	1.60 × 10^−2^
hsa00270	Cysteine and methionine metabolism	Amino acid	3	1.60 × 10^−2^
hsa00480	Glutathione metabolism	Amino acid	3	1.70 × 10^−2^
hsa00450	Selenocompound metabolism	Amino acid	2	1.70 × 10^−2^
hsa00910	Nitrogen metabolism	Energy	2	1.70 × 10^−2^
hsa00230	Purine metabolism	Nucleotide	4	1.90 × 10^−2^
hsa00532	Glycosaminoglycan biosynthesis	Glycan	2	2.26 × 10^−2^
hsa00650	Butanoate metabolism	Carbohydrate	2	3.00 × 10^−2^

**Table 2:** Quantification and comparison of the single-cell metabolic score across the cell types from human hepatoblastoma tumor samples: dataset GSE180665. *p*-values were obtained by a two-sided Student’s *t*-test.

Comparison	Mean_Tumor Cells	Mean Compared Group	*p*_Value
Tumor cells vs. Hepatocytes	0.120	−0.976	0.00 × 10^0^
Tumor cells vs. Hepatic stellate cell	0.120	0.012	3.49 × 10^−14^
Tumor cells vs. Edothelial cell	0.120	−0.214	0.00× 10^0^
Tumor cells vs. Kupffer cell	0.120	0.038	9.11 × 10^−27^
Tumor cells vs. B cell	0.120	−0.320	2.67 × 10^−9^
Tumor cells vs. Proliferative Hepatocyte	0.120	−1.250	1.68 × 10^−45^
Tumor cells vs. T NK cell	0.120	−0.400	4.05 × 10^−117^
Tumor cells vs. Cholangiocyte	0.120	−0.614	2.26 × 10^−53^
Tumor cells vs. Lymphatic	0.120	−0.256	2.17 × 10^−9^
Tumor cell vs. T_NK cell	0.120	0.016	8.26 × 10^−13^
Tumor cell vs. Neuronal cell	0.120	−0.195	2.56 × 10^−23^
Tumor cell vs. Erythrocyte	0.120	0.042	3.91 × 10^−4^

## Data Availability

The bioinformatics scripts used during this work are accessible at the following address: https://github.com/cdesterke/hepatoblastoma2024_script (accessed on 1 August 2024).

## Supplementary Materials

The following supporting information can be downloaded at: https://www.mdpi.com/article/10.3390/ijms252313044/s1.
