# Supplementary material for: Characterization of an Activated Metabolic Transcriptional Program in Hepatoblastoma Tumor Cells Using scRNA-seq
Source: Int J Mol Sci. 2024 Dec 4;25(23):13044. doi: 10.3390/ijms252313044 (PMC11641740; doi:10.3390/ijms252313044)
Supplement: Supplementary file 1 [file ijms-25-13044-s001.zip › ijms-3316991-supplementary.pdf]

Supplemental Material

Supplemental Figures

Figure S1: RNA-sequencing preprocessing of dataset GSE104766: A/voom transformation of the EdgeR normalized matrix; B/Denstiyplot by sample of the voom transformed data; C/Denstiyplot by sample after filtration of unexpressed genes; D/ Denstiyplot by sample after quantile normalization.

Figure S2: Harmonization of single transcriptome from human hepatoblastoma tissue samples: GSE180665, A/ Principal component analysis elbowplot of unintegrated single cell transcriptome data, B/ Unintegrated UMAP dimension reduction with original cell annotation, C/Unintegrated UMAP dimension reduction with sample origin annotation, D/ Dimension reduction post harmony integration with original cell annotation, E/Dimension reduction post harmony integration with sample origin annotation.

Figure S3: Single cell RNAseq preprocess for GSE180665 dataset: (A) UMAP dimension reduction post harmony with sample origin annotation (B) UMAP dimension reduction post harmony with group sample annotation (C) UMAP dimension reduction post harmony with original cluster annotation (D) UMAP dimension reduction post harmony with original cell annotation

Figure S4: Predictive values of activated metabolic markers in tumor cells from hepatoblastoma at single cell level: A/Elasticnet plot of fit with alpha fixed to 0.1; B/ ROC curve and area undercurve of the 41 metabolic markers with positive elasticnet coefficient for predicting tumor cell status in hepatoblastoma single cell transcriptome

Supplemental Table

Table S1: Differential expressed metabolic markers in human hepatoblastoma tumors (10.6084/m9.figshare.26413144) available at address: https://figshare.com/articles/dataset/Table_S1_Differential_expressed_metabolic_markers_in_human_hepatoblastoma_tumors/26413144?file=48042013 (accessed on 31 July 2024).

Figure S1

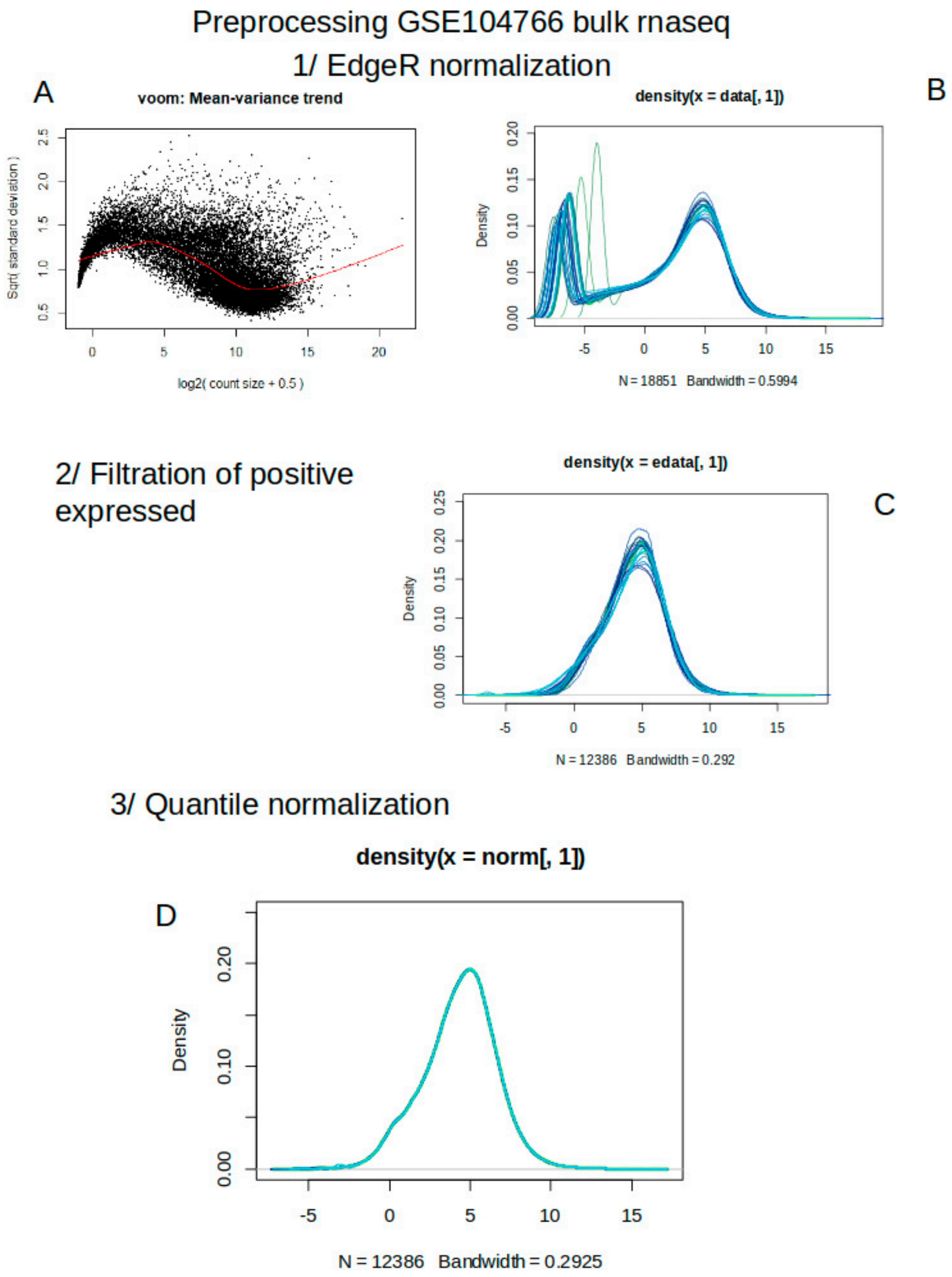

Preprocessing GSE104766 bulk rnaseq
1/ EdgeR normalization
A
voom: Mean-variance trend
Sqrt(standard deviation)
log2(count size + 0.5)
B
density(x = data[, 1])
Density
N = 18851 Bandwidth = 0.5994
2/ Filtration of positive expressed
C
density(x = edata[, 1])
Density
N = 12386 Bandwidth = 0.292
3/ Quantile normalization
D
density(x = norm[, 1])
Density
N = 12386 Bandwidth = 0.2925

Figure S2

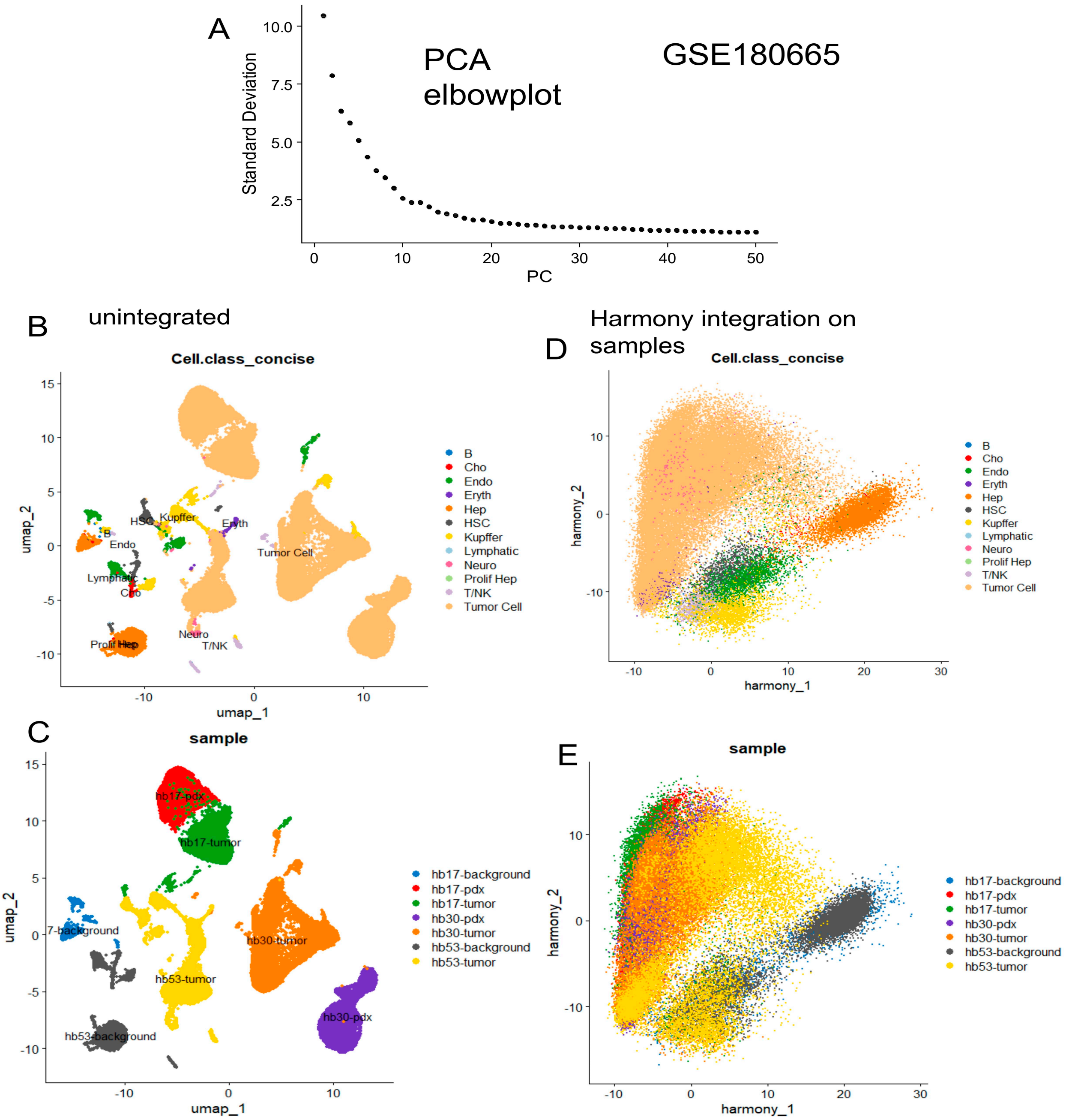
A
PCA
elbowplot
GSE180665
Standard Deviation
PC
B
unintegrated
Cell.class_concise
umap_1
umap_2
B
Cho
Endo
Eryth
Hep
HSC
Kupffer
Lymphatic
Neuro
Prolif Hep
T/NK
Tumor Cell
C
sample
hb17-background
hb17-pdx
hb17-tumor
hb30-pdx
hb30-tumor
hb53-background
hb53-tumor
D
Harmony integration on samples
Cell.class_concise
harmony_1
harmony_2
E
sample

Figure S3

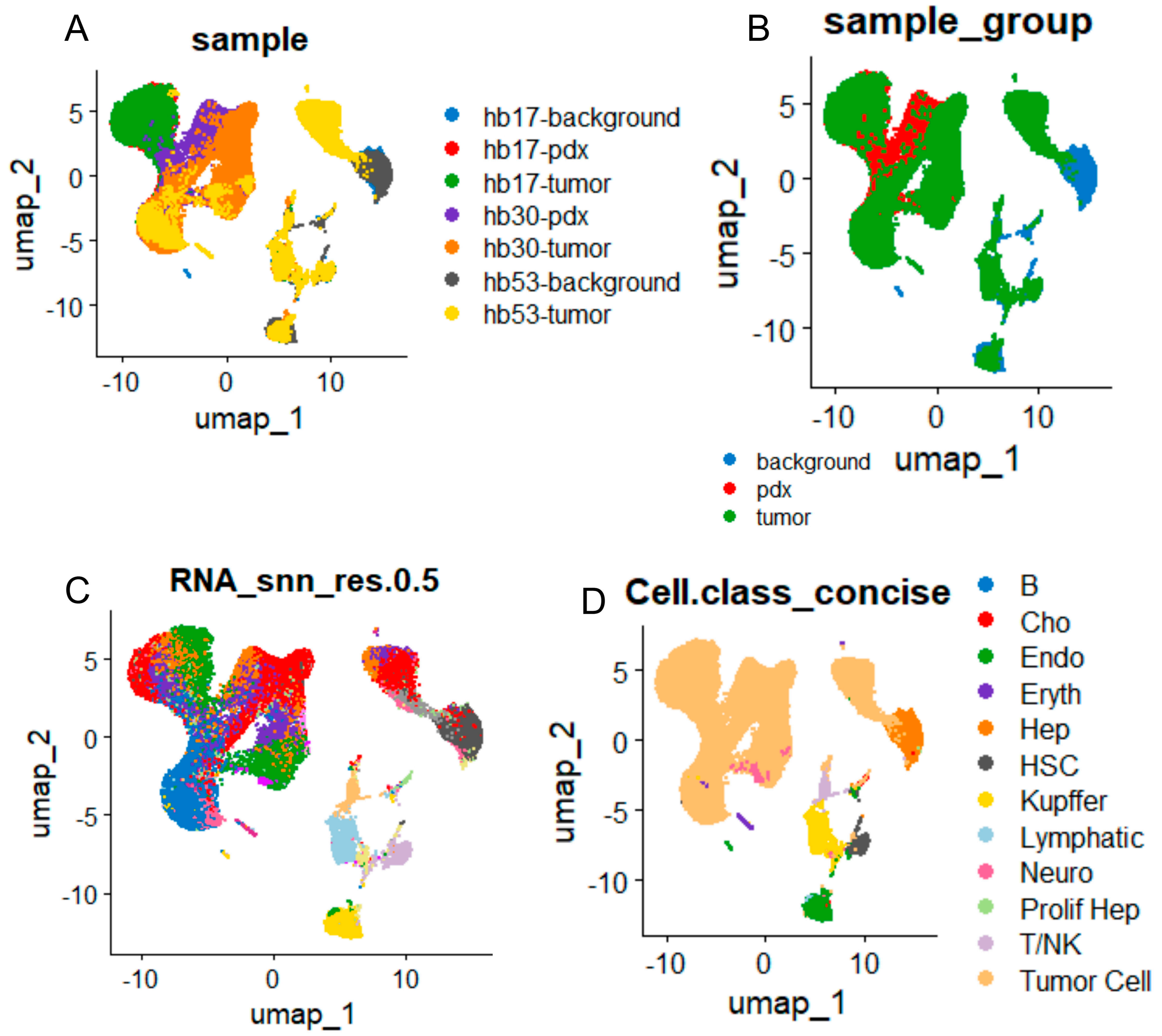
A
sample
umap_2
umap_1
hb17-background
hb17-pdx
hb17-tumor
hb30-pdx
hb30-tumor
hb53-background
hb53-tumor
B
sample_group
umap_2
umap_1
background
pdx
tumor
C
RNA_snn_res.0.5
umap_2
umap_1
D
Cell.class_concise
umap_2
umap_1
B
Cho
Endo
Eryth
Hep
HSC
Kupffer
Lymphatic
Neuro
Prolif Hep
T/NK
Tumor Cell

Figure S4

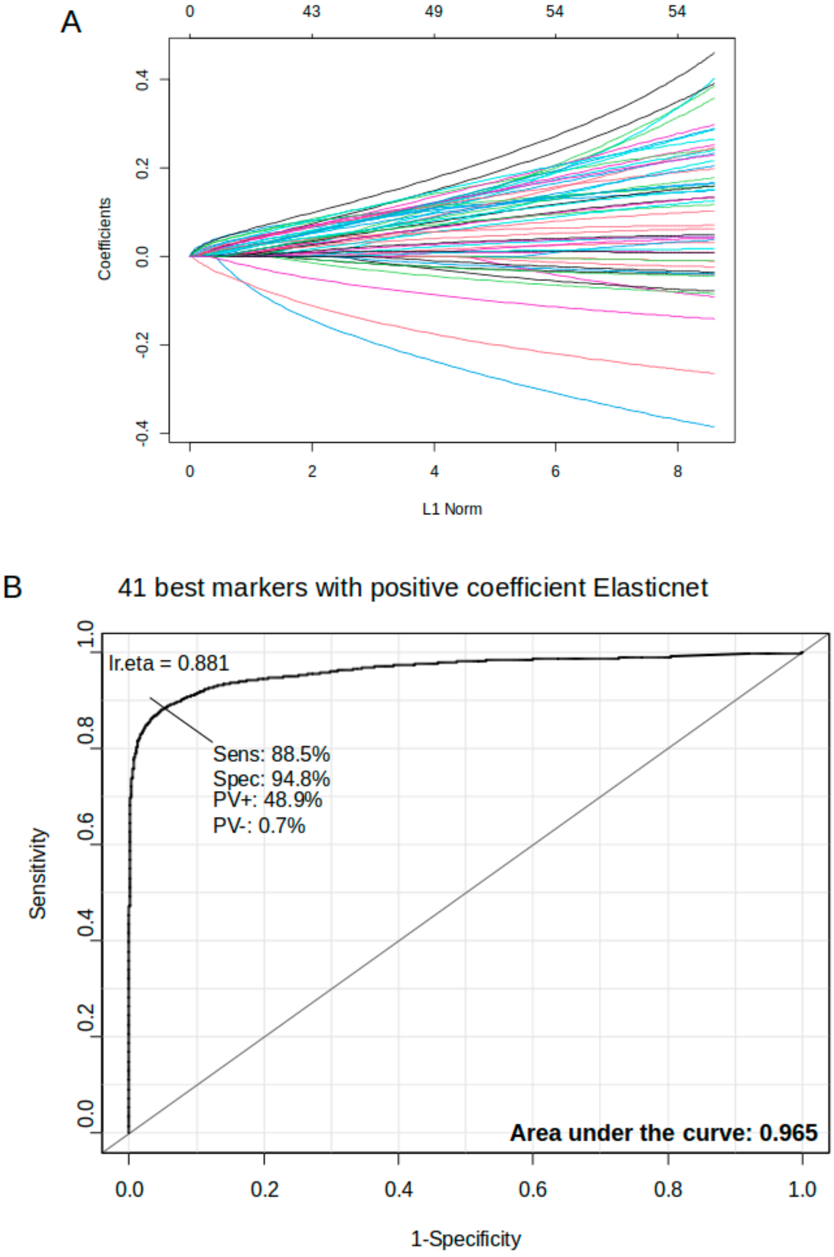
A
0
43
49
54
54
Coefficients
0.4
0.2
0.0
-0.2
-0.4
0
2
4
6
8
L1 Norm
B
41 best markers with positive coefficient Elasticnet
lr.eta = 0.881
Sens: 88.5%
Spec: 94.8%
PV+: 48.9%
PV-: 0.7%
Area under the curve: 0.965
Sensitivity
0.0
0.2
0.4
0.6
0.8
1.0
1-Specificity
0.0
0.2
0.4
0.6
0.8
1.0
